# Patient ambassador support in newly diagnosed patients with acute leukemia during treatment: a feasibility study

**DOI:** 10.1007/s00520-020-05819-w

**Published:** 2020-10-13

**Authors:** Kristina Holmegaard Nørskov, Dorthe Overgaard, Jannie Boesen, Anne Struer, Sarah Elke Weber Due El-Azem, Anders Tolver, Kirsten Lomborg, Lars Kjeldsen, Mary Jarden

**Affiliations:** 1 Department of Hematology, Rigshspitalet, Blegdamsvej 9, 2100 Copenhagen Ø, Denmark; 2 University College Copenhagen, Tagensvej 18, 2200 Copenhagen N, Denmark; 3 Department of Hematology, Herlev Hospital, Borgmester Ib Juuls Vej 1, 2730 Herlev, Denmark; 4 Department of Hematology, Zealand University Hospital, Roskilde, Sygehusvej 10, 4000 Roskilde, Denmark; 5 Data Science Laboratory, Department of Mathematical Sciences, University of Copenhagen, Universitetsparken 5, 2200 Copenhagen N, Denmark; 6 Department of Clinical Medicine, Aarhus University, Paalle Juul-Jensens Boulevard 82, 8200 Aarhus N, Denmark; 7 Department of Clinical Research, Steno Diabetes Center Copenhagen (SDCC), Niels Steensens Vej 2, 2820 Gentofte, Denmark; 8 Department of Clinical Medicine, University of Copenhagen, Blegdamsvej 3B, 2200 Copenhagen N, Copenhagen, Denmark

**Keywords:** Peer support, Patient ambassador, Acute leukemia, Supportive care, Feasibility, Psychosocial

## Abstract

**Purpose:**

This study investigated the feasibility of patient ambassador support in newly diagnosed patients with acute leukemia during treatment.

**Methods:**

A multicenter single-arm feasibility study that included patients newly diagnosed with acute leukemia (*n* = 36) and patient ambassadors previously treated for acute leukemia (*n* = 25). Prior to the intervention, all patient ambassadors attended a 6-h group training program. In the intervention, patient ambassadors provided 12 weeks of support for patients within 2 weeks of being diagnosed. Outcome measures included feasibility (primary outcome), safety, anxiety, and depression measured by the Hospital Anxiety and Depression Scale, quality of life by the Functional Assessment of Cancer Therapy–Leukemia and the European Organization for Research and Treatment of Cancer Quality of Life Questionnaire, and symptom burden by MD Anderson Symptom Inventory, the Patient Activation Measure, and the General Self-Efficacy Scale.

**Results:**

Patient ambassador support was feasible and safe in this population. Patients and patient ambassadors reported high satisfaction with the individually adjusted support, and patients improved in psychosocial outcomes over time. Patient ambassadors maintained their psychosocial baseline level, with no adverse events, and used the available support to exchange experiences with other patient ambassadors and to manage challenges.

**Conclusion:**

The patient ambassador support program is feasible and has the potential to be a new model of care incorporated in the hematology clinical care setting, creating an active partnership between patients and former patients. This may strengthen the existing supportive care services for patients with acute leukemia.

**Trial registration:**

NCT03493906

## Introduction

Acute leukemia (AL) is a malignant hematological disease with a rapid onset which, in curative treatment regimens, is followed by intensive high-dose chemotherapy, risk of life-threatening complications, and a significant symptom burden [1–5]. AL is classified into subtypes of acute myeloid or lymphoid leukemia (AML/ALL), each with distinguishing characteristics affecting both prognosis and treatment. A sub-type of AML is acute promyelocytic leukemia (APL) accounting for around 5–10% of all AML diagnosis [1]. Through the last decade, curative regimens for AL have only improved to a limited extent [1], while supportive care has improved significantly [6–9]. Specifically, in Northern Europe and the USA, an increasing number of patients are receiving the majority of their treatment in the outpatient setting [6–9]. These improvements are crucial but can result in the patients having less contact with health professionals and other patients with AL during treatment.

Being diagnosed with a life-threatening disease like AL, which comprises an unpredictable long-term clinical course, can be a traumatic experience, and many patients report high levels of psychological distress [2, 10–12]. In a previous study, we identified that newly diagnosed patients with AL experienced feeling jolted by the diagnosis and uncertainty about the future [13]. Moreover, they considered social support, including support from other patients with AL, as a lifeline because it had the potential to help them actively manage their situation and, more importantly, regain hope [13].

Peer support may benefit not only the person being supported but also the supporter [14, 15]. Peers possess an understanding and a first-hand experience of the disease and its treatment, and may provide support to a peer who is at an earlier stage of treatment or recovery [16]. Social comparison theory may partially explain the beneficial influence of peer support [17]. Comparisons with others in a similar situation to oneself can normalize the experience, provide positive role modeling, reduce the threat, and aid in coping with the new challenges [18]. In peer support programs, the peer supporter may also find comparisons helpful because they put their own disease trajectory and life experiences into perspective [14, 19]. The evidence of the effect of peer support programs in patients with cancer is growing [20, 21]. A review of one-to-one peer support programs in cancer care substantiates the beneficial effect on the psychosocial adjustment and the resulting high participant satisfaction with peer support [21]. Yet, due to the potential vulnerability of peer supporters, it is suggested that future research monitor their psychosocial state and elucidate the potential impact on patients and peer supporters [19, 21].

In one study, it has been shown that patients with AL requested support interventions in which former patients treated for AL support patients newly diagnosed with AL [22]. There is no evidence to date on the feasibility of a one-to-one peer support intervention in patients with AL [20, 21]. The existing research can only be transferred, to a limited extent, to patients with AL. Thus, due to acute onset, the intensity of treatment regimens often complicated by serious infections, and the risk of substantial symptom burden, it is relevant to investigate this type of social support in patients with AL. In the present study, a peer supporter is a former patient previously treated for AL who was named a patient ambassador (PA). This study was conducted to investigate the feasibility of patient ambassador support (PAS) in newly diagnosed patients with AL during initial treatment.

## Material and methods

### Study design

This multicenter single-arm feasibility study was conducted at three hematology departments in Denmark: Rigshospitalet, Herlev/Gentofte Hospital and Zealand University Hospital, Roskilde. The intervention included a 12-week PAS program for newly diagnosed patients with AL during their initial treatment with high-dose chemotherapy.

### Participants and procedures

The study included two categories of participants recruited from all three hematology departments: patients and PAs.

Eligibility criteria:

- Patients > 18 years and included within the first 2 weeks from time of diagnosis with acute myeloid leukemia or acute lymphatic leukemia if intensive chemotherapy treatment was planned.
- PAs > 18 years, previously diagnosed and treated for AL with intensive chemotherapy, at least 1 year since diagnosis, and in complete remission.

Participants were excluded if they did not understand, if they did not read and speak Danish, and if they had an unstable medical disease or any cognitive/psychiatric disorders.

#### Recruitment

PAs were recruited voluntarily from October 2017 to January 2018 using posters and flyers at the hematology departments (*n* = 4) and the Patient Association of Lymphoma, Leukemia, and Myelodysplastic Syndromes (*n* = 1), or they were selected and then approached by phone or mail (*n* = 30) by their primary hematologist in cooperation with the principle investigator (KHN), who screened eligible PAs for their suitability in a telephone interview. The PAs received a monetary incentive of 130 euro to cover transport expenses. The project nurse and primary investigator approached and recruited patients from February 2018 to June 2019 at the inpatient or outpatient clinic. Eligible participants received oral and written information from the principle investigator. Included participants then provided written informed consent prior to inclusion, and the PAs also signed a confidentiality agreement. Exclusion criteria for the participants were relapse (PAs), psychological conditions (delirium or severe depression), hospitalization in intensive care unit for more than 2 weeks, or transition to terminal care.

### Intervention

#### Preparation for the intervention

Prior to the intervention, the PAs attended a specially tailored 6-h program carried out by the principle investigator, the project nurse, and the project psychologist. The program included an introduction to the study, an overview of the disease and treatment regimes, and information and training on psychological issues and communication skills. There were discussions in small groups and in plenum on their personal goals, motivation, and concerns about volunteering. Upon completion of the training program, they received an information dossier with a checklist and guidelines, which included a list of relevant actions for PAs to take, and a tool to document the intervention.

#### PAS program

PAs provided 12 weeks of support to patients newly diagnosed with AL. Included patients and PAs were matched by the principle investigator immediately upon receipt of their informed content according to sex, age, type of AL, and/or other factors individually expressed prior to the intervention. Patients and ambassadors were matched independent of which of the three hematology departments they were recruited from. The PA initiated contact with the patient within 48 h, either by phone (conversation/text message), e-mail, or a face-to-face meeting, depending on the individual patient’s needs. However, face-to-face meetings were recommended for the purpose of developing a relationship. PAs followed one patient at a time, with a minimum of 4 weeks between patients. The primary investigator followed up on the initial and final contact, and during the intervention, if necessary.

#### Support and safety

During the intervention, the PAs were offered network meetings with supervision every 6 weeks with the principle investigator and the psychologist. If requested, the psychologist also provided individual supervision during the intervention.

### Outcome measures

#### Primary outcome

Feasibility studies focus on the process of developing and implementing the intervention, and eight areas of focus are described as feasibility criteria [23]. We adopted the following criteria in this study: acceptability, practicability, and safety and support [23, 24]. Evaluations were also obtained from patients and PAs. Finally, the PAs kept a record of the frequency, type, and topics of their communication. Participant and disease characteristics were obtained from the patient and PA, and from medical records.

#### Secondary outcome

Participants filled out electronic or paper versions of patient-reported outcome questionnaires at baseline and at the 12- and 24-week follow-up. Psychological well-being was assessed and measured using the Hospital Anxiety and Depression Scale (HADS) [25], while quality of life (QOL) was assessed using the Functional Assessment of Cancer Therapy–Leukemia (FACT-LEU) [26] and the European Organization for Research and Treatment of Cancer Quality of Life Questionnaire (EORTC QLQ-C30) [27]. Symptom burden was assessed using the MD Anderson Symptom Inventory (MDSAI) [28], while the Patient Activation Measure (PAM) [29, 30] was used to gauge the patients’ understanding of their own health and health care, and coping appraisal was assessed with the General Self-Efficacy Scale (GSE) [31].

### Statistical analysis

REDCap was used to collect and manage survey data and as an online record to register all contacts from participants with primary investigator, project nurses, and the psychologist [32, 33]. A sample size of 30 is recommended for feasibility trials. Due to the prognosis and significant symptom burden in patients with AL, they have a risk of high attrition, which is why we set a sample size of 35 in each group of participants [34]. The demographic and clinical characteristics of participants were summarized using numbers and percentages for categorical variables. PA characteristics were included once, regardless of the number of patients they followed. Follow-up data only contains data from participants who have completed the intervention. Patient-reported outcome measures were summarized using mean and standard deviation (SD). Official scoring manuals including guidelines for handling missing answers were used for computation of subscale scores. Data from one item of the FACT-LEU scale was not collected and is treated as a missing value for all participants when computing the subscale score. A linear mixed-effect model with random effect of participants and fixed effect of assessment time was used to analyze changes between baseline to 12-week follow-up and between the 12- and 24-week follow-up. The Wald test was used to test the hypothesis that changes equal to zero. *P* values < 0.05 were used to determine statistical significance, and the data analysis was carried out using IBM SPSS Statistics for Windows version 25 and R [35].

## Results

### Participant characteristics

In total, 36 patients and 24 PAs were included (Table 1). Females made up 58.3% of patients and 50% of PAS, while the age range was 21–77 (mean age, patients: 54.5 years; PAs: 51.5 years). PAs were slightly more frequently married or living with a partner compared to patients. Acute myeloid leukemia was the most frequent diagnosis in both patients (66.7%) and PAs (50.0%). A little less than half (44%) of the PAs were more than 4 years from their AL diagnosis, and 68% had undergone allogeneic hematopoietic stem cell transplantation.

**Table 1:** Demographic and clinical characteristics of study participants

Characteristic	Patients *N* = 36	Ambassadors *N* = 24
	Value	Value
Gender, female *n* (%)	21 (58.3)	12 (50.0)
Age, mean (range)	54.5 (27–77)	51.5 (21–76)
Education, *n* (%)		
No high school degree	4 (11.1)	1 (4.2)
High school degree	1 (2.7)	3 (12.5)
2 year in college	13 (36.1)	6 (25.0)
4 year in college	9 (25.0)	9 (37.5)
Master’s degree or higher	7 (19.4)	5 (20.8)
Occupation, *n* (%)		
Salaried employee	17 (47.2)	11 (45.8)
Unemployed	0	0
Retired employee	15 (41.7)	8 (33.3)
Sickness benefits	2 (5.6)	2 (8.3)
Undergoing education	2 (5.6)	3 (12.5)
Marital status, *n* (%)		
Married or cohabitating	25 (69.4)	19 (79.2)
Single, separated, divorced, or widowed	10 (27.8)	5 (20.8)
Unknown	1 (2.8)	0
Diagnosis, *n* (%)		
Acute lymphatic leukemia	11 (30.6)	8 (33.3)
Acute myeloid leukemia	24 (66.7)	12 (50.0)
Acute promyelocytic leukemia	1 (2.8)	4 (16.7)
Treatment, *n* (%)		
DA 3 + 10	18 (50.1)	
FLAG-IDA	3 (8.3)	
NOPHO	8 (22.2)	
Other	7 (19.4)	
Years post AL diagnosis, *n* (%)		
< 2		7 (29.2)
2–4		7 (29.2)
> 4		10 (41.6)
Allogeneic HSCT, *n* (%)		16 (66.6)
Years post HSCT, *n* (%)		
< 2		7 (43.7)
2–4		3 (18.7)
> 4		6 (37.5)

*DA 3 + 10*, daunorubicin-Ara-C; *FLAG-IDA*, fludarabine, cytarabine, idarubicin, and G-CSF; *NOPHO*, Nordic Society of Pediatric Haematology and Oncology; *HSCT*, hematopoietic stem cell transplantation

### Feasibility criteria

#### Acceptability

**Fig. 1:**
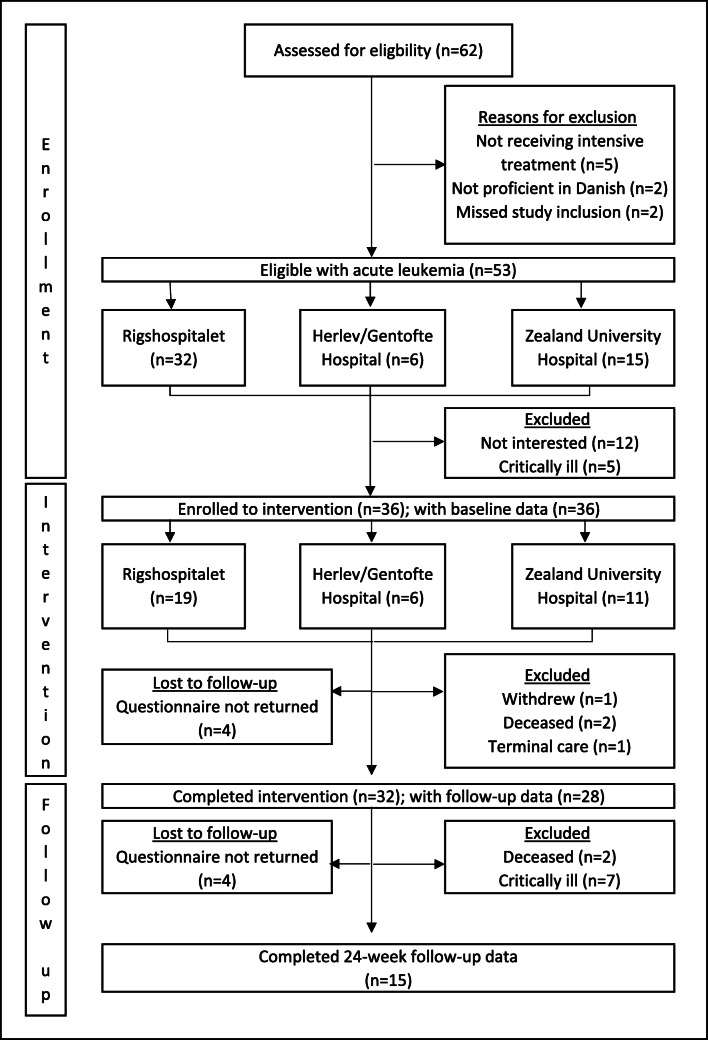
Flowchart on patients

**Fig. 2:**
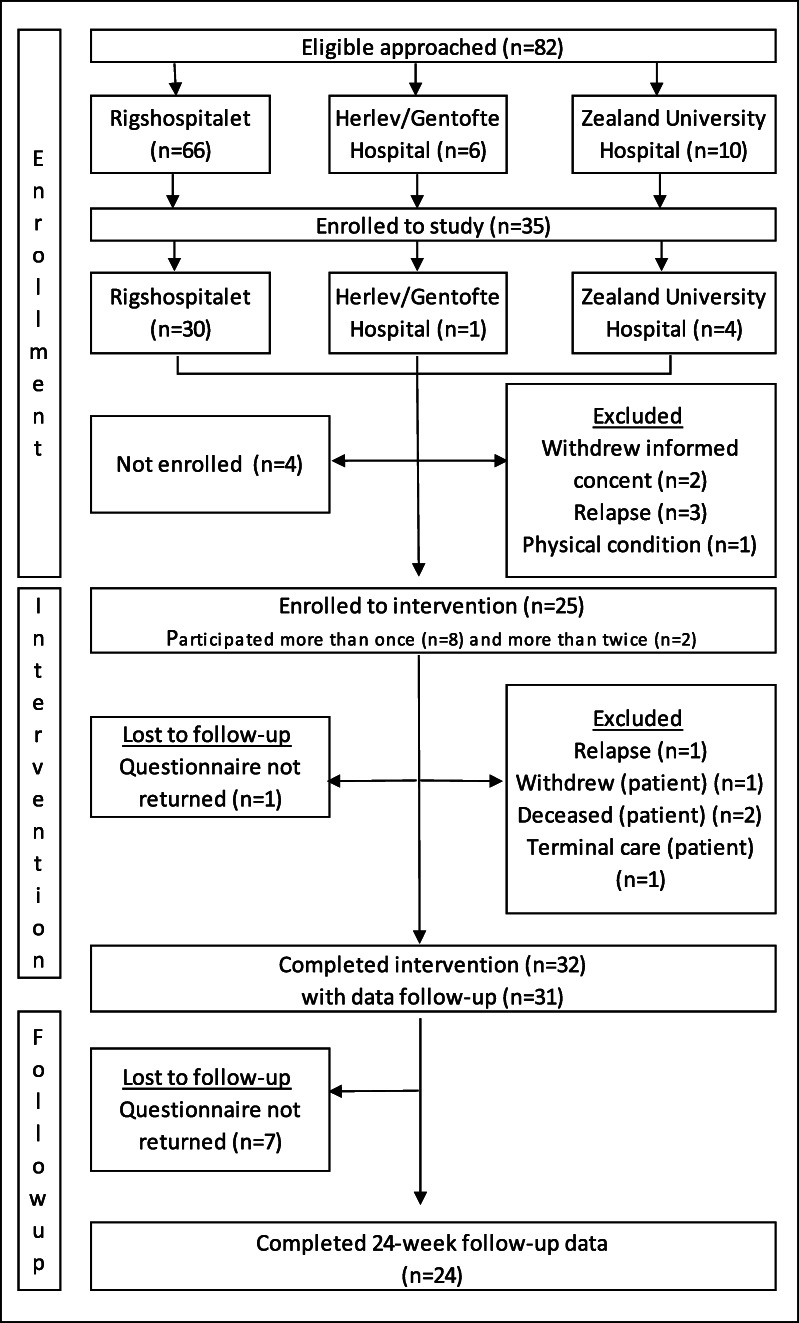
Flowchart on patient ambassadors

A total of 53 eligible patients were approached (Fig. 1), 36 of whom were accepted for participation, and 17 of whom declined participation, mainly due to the following: a lack of physical and/or psychological strength to participate; already had enough support from own network; comorbidities; did not want to become immersed in their own disease; and did not want to involve unfamiliar parties in the course of their disease and treatment. Four patients were lost to follow-up due to transition to terminal care (*n* = 1), death (*n* = 2), and withdrawal (*n* = 1). In total, 32 patients completed the intervention. A total of 82 eligible PAs were approached (Fig. 2), 35 of whom agreed to participate, and 25 of whom were enrolled in the intervention. After enrollment, six PAs were lost to follow-up due to relapse, their patient died, was transferred to terminal care, or withdrew. In total, 24 PAs completed the intervention, and 12 participated more than once. Patients and PAs were largely satisfied with the intervention, with 96.3% of patients (*n* = 27) and 80.6% of PAs (*n* = 31) reporting a satisfaction level ≥ 5 out of 10. The intervention also had an acceptable influence on the patient’s disease and treatment trajectory, with 74.0% reporting ≥ 5 out of 10 points.

#### Practicability

All 35 enrolled PAs participated in the mandatory educational 6-hour program prior to the intervention. The PA course was reported useful (86.6%) in relation to what they experienced, and the majority (93.3%) reported receiving enough information and knowledge about their new role. Throughout the intervention, 10 network meetings were held, with participation at each meeting reaching three to 13 PAs.

**Fig. 3:**
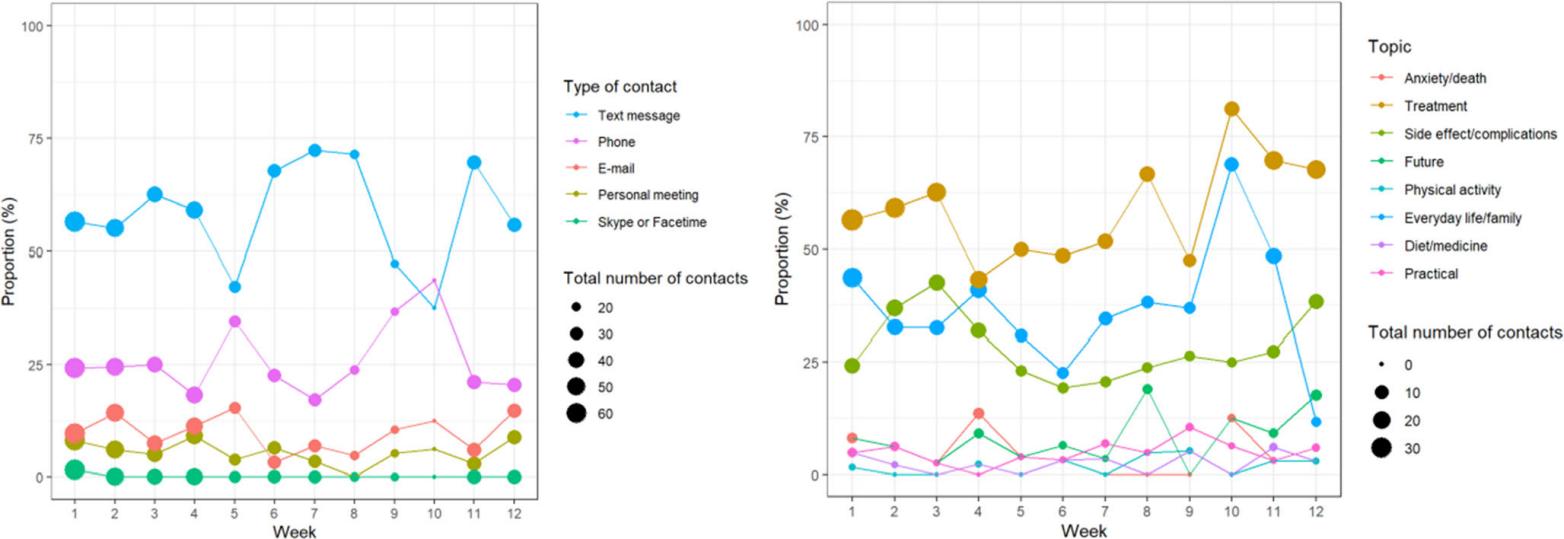
Contacts between participants

Meeting personally with patients was challenging, primarily because of the patients’ lack of strength, hospitalization, reduced immune system, many visits from their own social network, or geographical distance. Only 9.3% had four personal meetings during the intervention, 3.1% had three meetings, 3.1% had two, 21.9% one, and 62.5% none. There were 404 contacts between patients and PAs, with a mean of 12.6 contacts per dyad. The number of contacts was decreasing during the intervention, with a small increase at the end of the period (Fig. 3). Our data shows that text messages and telephone conversations were used the most to make contact. Figure 3 provides an overview of the distribution of conversation topics between participants during the intervention, with treatment the most common, followed by side effects, complications, everyday life, and family.

#### Safety and support

None of the PAs needed individual support from the project psychologist, and they only initiated contact with health professionals during the intervention. There were 16 PAs who initiated contact with the principle investigator, interspersed as follows: one contact (*n* = 7), two contacts (*n* = 2), three contacts (*n* = 3), four contacts (*n* = 2), and six contacts (*n* = 1). Reasons for contact were evaluation of initiating the relation; challenges in establishing the relationship; death of patient; and patient unsure of whether to stay in the intervention. PAs primarily found support in network meetings (76.5%), principle investigator (23.5%), and their spouse (17%). Reasons for seeking support were the need to talk with others and hear their experiences with the role of PA; managing challenges in establishing the relationship with the patient; and coping when the patient’s treatment failed. One patient ambassador experienced a relapse during the intervention, causing the patient concern because the patient ambassador functioned as a beacon of hope for the future. The worry did not persist, and the patient and principle investigator jointly decided that she did not need further support. However, the new circumstances meant that they maintained contact and took a more equal role. No unexpected adverse events occurred during the intervention.

### Clinical outcome

We studied multiple patient-reported outcome variables, which are listed for patients in Table 2 and for PAs in Table 3. An overall trend showed that patients improved in all sum scores over time, from baseline to week 24. The patient’s mean score was above the cutoff score (> 8) for anxiety at baseline, but improved by 12-week follow-up, scoring below the cutoff point. For patients, statistically significant improvements from baseline to 12-week follow-up were found for anxiety (*p* = 0.007), global health (*p* = 0.047), role functioning (*p* = 0.014), cognitive functioning (*p* = 0.044), functional well-being (*p* = 0.014), and patient activation level (*p* = 0.021). Conversely, PAs did not change significantly over time in any of the clinical outcomes, with the exception of emotional well-being (*p* = 0.004) from baseline to 12-week follow-up.

**Table 2:** Patient-reported outcomes in patients

Variables	Baseline (*N* = 36)		12-week follow-up (*N* = 28)		24-week follow-up (*N* = 15)		Baseline to 12-week follow-up			12-week to 24-week follow-up		
	*N*	Mean (SD)	*N*	Mean (SD)	*N*	Mean (SD)	Change (s.e.)	95% CI	*P* value	Change (s.e.)	95% CI	*P* value
HADS												
Anxiety (0–21)	*35*	8.2 (3.8)	*28*	6.2 (4.4)	*15*	6.8 (3.7)	*´*− 2.1 (0.7)	[− 3.6; − 0.6]	0.01*	0.0 (0.9)	[− 1.8; 1.9]	0.98
Depression (0–21)	*35*	7.3 (4.3)	*28*	5.5 (3.9)	*15*	6.2 (4.5)	´− 1.3 (0.7)	[− 2.7; 0.1]	0.07	0.7 (0.9)	[− 1.1; 2.5]	0.46
MDASI												
Core (0–10)	*35*	3.5 (1.9)	*27*	3.1 (2.5)	*14*	3.7 (2.2)	´− 0.3 (0.5)	[− 1.2; 0.6]	0.52	0.7 (0.6)	[− 0.4; 1.9]	0.21
Interference (0–10)	*35*	3.7 (2.5)	*28*	3.4 (3.0)	*14*	3.5 (2.6)	´− 0.1 (0.6)	[− 1.3; 1.0]	0.85	0.5 (0.7)	[− 1.0; 1.9]	0.54
EORTC QLQ-C30												
Global health (0–100)	*35*	40.5 (22.5)	*27*	55.6 (27.8)	*14*	61.3 (30.4)	11.5 (5.6)	[0.1; 22.8]	0.05*	6.0 (7.2)	[− 8.5; 20.5]	0.41
Physical functioning (0–100)	*33*	67.5 (24.4)	*28*	70.5 (23.5)	*14*	71.9 (23.7)	´− 1.6 (5.0)	[− 11.7; 8.5]	0.76	0.6 (6.3)	[− 12.2; 13.4]	0.93
Role functioning (0–100)	*31*	28.5 (31.4)	*28*	51.2 (34.5)	*13*	57.7 (33.8)	20.8 (8.1)	[4.4; 37.2]	0.01*	5.0 (10.2)	[− 15.8; 25.7]	0.63
Emotional functioning (0–100)	*34*	71.5 (21.5)	*28*	74.1 (21.2)	*14*	71.6 (22.5)	0.9 (3.8)	[− 6.9; 8.8]	0.81	´− 1.5 (4.9)	[− 11.5; 8.5]	0.76
Cognitive functioning (0–100)	*34*	72.5 (21.3)	*28*	81.0 (18.0)	*14*	82.1 (19.0)	9.0 (4.3)	[0.3; 17.8]	0.04*	´− 0.4 (5.5)	[− 11.6; 10.8]	0.94
Social functioning (0–100)	*34*	53.9 (36.0)	*28*	63.1 (28.5)	*14*	66.7 (39.2)	9.9 (6.8)	[− 3.9; 23.6]	0.15	´− 0.9 (8.7)	[− 18.5; 16.7]	0.92
FACT-LEU												
Physical well-being (0–28)	*34*	17.6 (5.6)	*28*	19.6 (7.1)	*14*	18.6 (8.4)	1.6 (1.4)	[− 1.3; 4.6]	0.26	´− 1.2 (1.9)	[− 5.0; 2.5]	0.51
Social/family well-being (0–28)	*34*	21.9 (4.1)	*28*	21.2 (5.3)	*14*	21.7 (5.6)	´− 0.6 (0.6)	[− 1.8; 0.7]	0.35	0.4 (0.8)	[− 1.2; 1.9]	0.66
Emotional well-being (0–24)	*35*	16.1 (4.8)	*28*	16.8 (4.3)	*14*	16.6 (5.2)	0.2 (0.7)	[− 1.3; 1.7]	0.76	0.8 (1.0)	[− 1.1; 2.8]	0.4
Functional well-being (0–28)	*34*	11.2 (5.8)	*28*	25.5 (6.7)	*13*	16.6 (8.6)	3.5 (1.4)	[0.8; 6.3]	0.01*	0.6 (1.8)	[− 3.0; 4.3]	0.73
FACT-G (0–108)	*33*	66.9 (15.9)	*28*	73.1 (18.2)	*13*	74.0 (23.3)	4.1 (3.0)	[− 2.0; 10.1]	0.18	0.1 (4.0)	[− 7.9; 8.2]	0.97
Leu subscale (0–68)	*35*	42.9 (11.7)	*28*	47.4 (9.2)	*14*	44.5 (12.3)	3.5 (2.2)	[− 1.0; 7.9]	0.13	´− 2.6 (2.9)	[− 8.4; 3.2]	0.36
FACT-LEU scale (0–176)	*33*	110.1 (26.7)	*28*	120.5 (26.4)	*13*	118.9 (34.8)	7.2 (5.0)	[− 3.0; 17.3]	0.16	´− 2.9 (6.6)	[− 16.3; 10.4]	0.66
TOI (0–124)	*33*	72.1 (21.6)	*28*	82.5 (21.2)	*13*	80.2 (28.6)	8.0 (4.6)	[− 1.3;17.3]	0.09	´− 3.9 (6.1)	[− 16.1; 8.4]	0.53
PAM												
Sum score (13–52)	*31*	37.6 (4.5)	*24*	40.1 (6.7)	*14*	39.0 (6.7)	2.2 (1.3)	[− 0.5; 5.0]	0.11	´− 0.4 (1.6)	[− 3.7; 3.0]	0.82
Niveau (1–4)	*31*	2.2 (1.0)	*24*	2.9 (0.9)	*14*	2.4 (1.2)	0.6 (0.3)	[0.1; 1.1]	0.02*	´− 0.4 (0.3)	[− 1.0; 0.3]	0.25
GSE												
Average score (1–4)	*34*	2.8 (0.6)	*28*	3.0 (0.7)	*13*	2.8 (0.7)	0.2 (0.1)	[− 0.1; 0.4]	0.22	´− 0.1 (0.2)	[− 0.5; 0.2]	0.37

Any available data from patients who did not complete the intervention is included in baseline summaries

*N*: number of particpants included in statistical analysis

*HADS*, Hospital Anxiety and Depression Scale, a 14-item measure with higher scores indicating higher symptomatology (cutoff scores > 8 for each item); *MDASI*, MD Anderson Symptom Inventory, a 19-item measure and assesses the severity of 13 symptoms and their impact in cancer patients; *EORTC QLQ-C30*, European Organisation for Research and Treatment of Cancer Quality of Life Questionnaire, a 30-item measure; *FACT-LEU*, Functional Assessment of Cancer Therapy–Leukemia, a 44-item measure; *FACT-G*, physical, social/family, emotional, and functional well-being; *FACT-LEU*, FACT-G and Leu subscale; *TOI*, trial outcome index: physical, functional well-being, and Leu subscale; *PAM*, Patient Activation Measure, a 13-item measure, with sum scores graded into PAM levels 1–4, with higher levels indicating better trust and competencies to cope; *GSE*, General Self-Efficacy Scale, a 10-item measure, with higher scores indicating greater sense of self-efficacy, range of score listed after each variable; *SD*, standard deviation; *s.e.*, standard error; *95% CI*, 95% confidence interval

*p < 0.05

**Table 3:** Patient-reported outcomes in patient ambassadors

Variables	Baseline (*N* = 36)		12-week follow-up (*N* = 31)		24-week follow-up (*N* = 24)		Baseline to 12-week follow-up			12-week to 24-week follow-up		
	*N*	Mean (SD)	*N*	Mean (SD)	*N*	Mean (SD)	Change (s.e.)	95% CI	*P* value	Change (s.e.)	95% CI	*P* value
HADS												
Anxiety (0–21)	*36*	3.6 (2.4)	*31*	3.1 (2.9)	*24*	3.3 (3.0)	´− 0.4 (0.5)	[− 1.4; 0.7]	0.46	0.4 (0.6)	[− 0.7; 1.5]	0.51
Depression (0–21)	*36*	2.0 (1.7)	*31*	2.0 (2.1)	*24*	2.0)2.1)	´− 0.1 (0.3)	[− 0.7; 0.5]	0.67	0.2 (0.3)	[− 0.5; 0.9]	0.6
MDASI												
Core (0–10)	*36*	1.5 (1.5)	*31*	1.8 (1.8)	*23*	1.4 (1.6)	0.4 (0.3)	[− 0.2; 1.0]	0.18	´− 0.5 (0.3)	[− 1.1; 0.2]	0.16
Interference (0–10)	*36*	1.4 (2.0)	*31*	1.4 (2.0)	*23*	0.8 (1.3)	´− 0.1 (0.4)	[− 0.9; 0.6]	0.73	´− 0.6 (0.4)	[− 1.5; 0.2]	0.14
EORTC QLQ-C30												
Global health (0–100)	*36*	80.3 (15.6)	*31*	78.2 (19.1)	*23*	80.8 (18.0)	´− 1.4 (2.2)	[− 5.8; 3.1]	0.54	1.1 (2.5)	[− 3–8; 6.1]	0.66
Physical functioning (0–100)	*36*	85.6 (20.8)	*31*	86.1 (17.2)	*23*	87.5(16.9)	1.3 (1.9)	[− 2.5; 5.1]	0.51	1.56 (2.1)	[− 2.8; 5.8]	0.48
Role functioning (0–100)	*36*	87.5 (20.8)	*31*	84.9 (20.8)	*23*	82.6 (25.4)	´− 0.6 (3.1)	[− 6.7; 5.5]	0.85	´− 2.3 (3.5)	[− 9.2; 4.6]	0.51
Emotional functioning (0–100)	*36*	90.7 (13.3)	*31*	92.5 (12.8)	*23*	96.0 (7.9)	1.9 (2.3)	[− 2.7; 6.7]	0.4	2.2 (2.6)	[− 3.0; 7.5]	0.4
Cognitive functioning (0–100)	*36*	87.0 (18.3)	*31*	84.4 (19.7)	*23*	86.2 (17.9)	´− 2.1 (1.9)	[− 6.1; 1.8]	0.29	1.2 (2.2)	[− 3.3; 5.7]	0.61
Social functioning (0–100)	*36*	86.6 (19.8)	*31*	88.2 (17.8)	*23*	87.7 (20.9)	1.5 (2.8)	[− 4.1; 7.2]	0.59	0.1 (3.2)	[− 6.2; 6.5]	0.97
FACT-LEU												
Physical well-being (0–28)	*36*	24.6 (3.0)	*31*	24.3 (3.9)	*23*	24.9 (3.7)	´− 0.1 (0.5)	[− 1.1; 0.9]	0.89	0.6 (0.6)	[− 1.1; 0.9]	0.89
Social/family well-being (0–28)	*36*	22.3 (4.4)	*31*	22.4 (4.7)	*23*	21.0 (6.4)	0.1 (0.8)	[− 1.5; 1.7]	0.9	´− 1.4 (0.9)	[− 3.1; 0.4]	0.12
Emotional well-being (0–24)	*36*	21.4 (2.5)	*31*	20.8 (2.5)	*23*	21.1 (2.3)	´− 1.0 (0.3)	[− 1.6; − 0.3]	<0.01*	0.4 (0.4)	[− 0.3; 1.1]	0.26
Functional well-being (0–28)	*35*	23.0 (4.7)	*31*	23.1 (4.4)	*23*	22.3 (6.3)	0.1 (0.6)	[− 1.2; 1.3]	0.93	´− 1.0 (0.7)	[− 2.4; 0.4]	0.15
FACT-G (0–108)	*35*	91.3 (11.8)	*31*	90.5 (11.5)	*23*	89.4 (16.2)	´− 1.1 (1.6)	[− 4.3; 2.1]	0.51	´− 1.3 (1.8)	[− 5.0; 2.3]	0.46
Leu subscale (0–68)	*36*	59.1 (6.8)	*31*	59.0 (6.2)	*23*	59.1 (8.3)	´− 0.4 (0.9)	[− 2.1; 1.4]	0.67	´− 0.1 (1.0)	[− 2.0; 1.9]	0.96
FACT-LEU (0–176)	*35*	150.3 (17.5)	*31*	150.0 (17.0)	*23*	148.5 (23.4)	´− 1.6 (2.0)	[− 5.7; 2.5]	0.43	´− 1.4 (2.3)	[− 6.0; 3.2]	0.54
TOI (0–124)	*35*	106.6 (12.6)	*31*	106.4 (12.5)	*23*	106.3 (16.7)	´− 0.7 (1.5)	[− 3.6; 2.3]	0.65	´− 0.5 (1.7)	[− 3.8; 2.9]	0.78
PAM												
Sum score (13–52)	*36*	44.0 (7.6)	*28*	44.1 (7.5)	*23*	45.3 (4.8)	0.1 (1.4)	[− 2.7; 2.8]	0.96	2.0 (1.5)	[− 1.1; 5.1]	0.2
Niveau (1–4)	*36*	3.3 (1.0)	*28*	3.3 (1.0)	*23*	3.6 (0.6)	0.1 (0.2)	[− 0.3; 0.5]	0.74	0.3 (0.2)	[− 0.1; 0.7]	0.16
GSE												
Sum score (1–4)	*36*	3.4 (0.5)	*31*	3.4 (0.6)	*23*	3.4 (0.6)	´− 0.0 (0.1)	[− 0.2; 0.1]	0.61	0.0 (0.1)	[− 0.1; 0.2]	0.66

Any available data from patients who did not complete the intervention is included in baseline summaries

*N*: number of particpants included in statistical analysis

*HADS*, Hospital Anxiety and Depression Scale, a 14-item measure with higher scores indicating higher symptomatology (cutoff scores > 8 for each item); *MDASI*, MD Anderson Symptom Inventory, a 19-item measure and assesses the severity of 13 symptoms and their impact in cancer patients; *EORTC QLQ-C30*, European Organisation for Research and Treatment of Cancer Quality of Life Questionnaire, a 30-item measure; *FACT-LEU*, Functional Assessment of Cancer Therapy–Leukemia, a 44-item measure; *FACT-G*, physical, social/family, emotional, and functional well-being; *FACT-LEU*, FACT-G and Leu subscale; *TOI*, trial outcome index: physical, functional well-being, and Leu subscale; *PAM*, Patient Activation Measure, a 13-item measure, with sum scores graded into PAM levels 1–4, with higher levels indicating better trust and competencies to cope; *GSE*, General Self-Efficacy Scale, a 10-item measure, with higher scores indicating greater sense of self-efficacy, range of score listed after each variable; *SD*, standard deviation; *s.e.*, standard error; *95% CI*, 95% confidence interval

*p < 0.05

## Discussion

### Discussion of results

To our knowledge, this is the first study to investigate a one-to-one peer support intervention in newly diagnosed patients with AL. The findings demonstrate that PAS was feasible and safe in this population, with high acceptability and satisfaction among both patients and PAs. However, there were challenges related to the wide amount of variation in how the support was provided, and in terms of the high disease and treatment-related symptom burden, emphasizing the importance of individualizing support in clinical practice. Support for the PAs was an indispensable aspect of the PAS program. Likewise, our qualitative evaluation of PAS showed that patients experienced a feeling of being understood, a cohesive relationship leading to hope and a feeling of being able to cope with their situation. Simultaneously, patient ambassadors experienced a sense of meaningfulness and gratitude for life [15].

This study demonstrated that PAS can be conducted in patients with AL undergoing intensive chemotherapy. Similar to other studies exploring peer support in cancer populations, we found the intervention to be acceptable, with high satisfaction among both patients and PAs [20, 21]. This may be explained by the benefits of social comparison processes, which play a pivotal role in understanding how people interpret health threats, understand their own health risks, and adapt to serious illness [18]. People facing a life-threatening disease may be compelled to use comparison as a way to counteract these issues [36]. Studies have revealed that patients with cancer prefer contact with, and information about, other cancer patients whose health is better than their own [37–39]. This upward social comparison may positively impact newly diagnosed patients during peer support because they can clarify what has happened (and is happening) to them, be assured by those who have survived the disease and treatment, and share their experiences with others [37–39].

In contrast, PAs may use downward comparisons to put their own disease trajectory into perspective by evaluating themselves against those perceived to be in poorer health, in this case the patients [36]. Regardless, if the difference between people is too significant, it may result in alienation, with no possibility of comparison [18]. Therefore, matching in peer support interventions is of great importance to achieve successful comparison between two peers. In our study, we matched participant preferences as closely as possible, which may explain the low dropout rate and the high satisfaction among both groups of participants.

Our results showed that patients improved over time in most psychosocial outcomes, which is consistent with other longitudinal studies examining QOL and psychological health in patients with AL throughout the treatment trajectory [40–42]. Although scores improved over time, the results were still significantly lower compared to normative data [43]. This highlights the importance of developing and undertaking interventions that improve QOL and psychosocial outcomes in patients with AL. Interestingly, PAs who maintained their psychosocial origin had QOL levels that were equal to or better than normative data [43]. This indicates two important perspectives to recognize in peer support interventions. First, PAs may benefit from their role as a peer supporter, and the role becomes a part of their own long-term psychological recovery. This has been confirmed in previous studies where peer supporters achieve a positive impact by putting their own disease trajectory and life experiences into perspective [14, 19, 21]. Second, PAs represent a selected group of peers who are psychologically robust, which is important as those who wish to participate are best suited for the role of peer supporter.

Several systematic reviews have examined the impact of peer support in cancer populations [20, 21, 44, 45]. However, depending on the cancer population, there is contradictory evidence on the provision of peer support [20]. Our results suggest that PAS in patients with AL should be provided individually as patients have different needs that change over time, depending on their disease trajectory and symptom burden. These results are in line with the general perspective of patient-centered care, which focuses on the individual’s particular health care needs and preferences [46].

Due to the peer supporter’s history of cancer and thus risk of increased vulnerability, monitoring their psychosocial status is imperative [20]. Our results demonstrate that psychosocial status in PAs does not change over time during their role as peer supporters, and none of the PAs took advantage of the opportunity to speak individually with the psychologist. This result should be viewed in the light of the tremendous effort that was put into preparing and supporting the PAs throughout the intervention. In line with this, a qualitative study (2013) exploring the experiences of peer supporters found no adverse consequences but emphasized the importance of providing support and training [19].

There is an indication that peer supporters perceive their support as being less effective and supportive than the peer support recipients did [45]. This potential discrepancy may explain why the PAs in the present study were less satisfied compared to the patients. Similar results were found in a previous qualitative study exploring the experiences of cancer patients and their peer supporters that showed that peer supporters found it challenging to strike the right balance between their own need to help and the patient’s need for help [14]. In a recent qualitative study, the motivation of PAs was explored and showed that their own disease course became meaningful, which facilitated a better recovery [47]. Therefore, taking their motivation and potential challenges into account is essential when training peer supporters.

### Discussion of methods

The strengths of this study include the longitudinal design and inclusion of three centers, with close monitoring of feasibility and the psychosocial well-being of all participants. Limitations include that participants were primarily not living alone and were well-educated, which may limit the representativeness of our findings. Patient demographic data on non-participants was not collected, which is why we cannot confirm their comparability. However, only a small number of patients declined participation due to having a sufficient social network. We encountered missing data at 24 weeks, mostly in patients, although this was expected to some degree due to their prognosis and significant symptom burden. This may have led to an overestimation of the sum scores at this time point.

### Clinical implications

Based on our results, we recommend PAS as a supplement to the existing supportive care service available to patients with AL. The PAs are not educated health care professionals, which is why it is essential that they receive the necessary education and support organized by an established network with collaboration between PAs, hospitals, and departments. Evidence is lacking on the timing, type, and duration of peer support, though many studies have assessed outcome measures such as coping, QOL, and psychological states without finding significant effects [20, 21]. This might suggest that these outcomes are not appropriate for assessing the effectiveness of peer support, and more immediate outcomes such as availability of social support could be more applicable in future research. Finally, the evidence to date is based on an examination of peer support provided either face-to-face, by telephone or as a group support. Our findings highlight the importance of providing individual support, and taking this approach is imperative to obtain high representativeness to initiate meaningful support that accommodates a broad group of patients.

## Conclusion

This study demonstrates that PAS in newly diagnosed patients with AL during initial treatment was feasible and safe. Patients and PAs reported high satisfaction with individual peer support, and patients’ psychosocial outcomes improved over time. PAs maintained psychosocial baseline levels, with no adverse events, and used the available support to exchange experiences with other PAs. The findings of this study have the potential to have an impact on psychosocial supportive care in patients with AL by informing the development of integrated psychosocial interventions. Our results are based on a sample of participants with AL, and future research is needed to confirm these results in patients and survivors with other hematological malignancies and cancers.
